# Patent Keyword Extraction Algorithm Based on Distributed Representation for Patent Classification

**DOI:** 10.3390/e20020104

**Published:** 2018-02-02

**Authors:** Jie Hu, Shaobo Li, Yong Yao, Liya Yu, Guanci Yang, Jianjun Hu

**Affiliations:** 1 Key Laboratory of Advanced Manufacturing Technology of Ministry of Education, Guizhou University, Guiyang 550025, China; 2 Department of Computer Science and Engineering, University of South Carolina, Columbia, SC 29208, USA; 3 School of Mechanical Engineering, Guizhou University, Guiyang 550025, China

**Keywords:** keyword extraction, information gain, patent classification, deep learning

## Abstract

Many text mining tasks such as text retrieval, text summarization, and text comparisons depend on the extraction of representative keywords from the main text. Most existing keyword extraction algorithms are based on discrete bag-of-words type of word representation of the text. In this paper, we propose a patent keyword extraction algorithm (PKEA) based on the distributed Skip-gram model for patent classification. We also develop a set of quantitative performance measures for keyword extraction evaluation based on information gain and cross-validation, based on Support Vector Machine (SVM) classification, which are valuable when human-annotated keywords are not available. We used a standard benchmark dataset and a homemade patent dataset to evaluate the performance of PKEA. Our patent dataset includes 2500 patents from five distinct technological fields related to autonomous cars (GPS systems, lidar systems, object recognition systems, radar systems, and vehicle control systems). We compared our method with Frequency, Term Frequency-Inverse Document Frequency (TF-IDF), TextRank and Rapid Automatic Keyword Extraction (RAKE). The experimental results show that our proposed algorithm provides a promising way to extract keywords from patent texts for patent classification.

## 1. Introduction

Patents are an important part of intellectual property. Effective patent analysis may bring lots of benefits for the enterprise. According to the analyzed objects, patent mining can be divided into patent metadata mining and patent text mining, between which the former is much more mature in terms of the methodology and analysis techniques. However, novel technological information is hidden in the descriptive text of patents. One of the main patent mining tasks is patent classification. In practical situations, usually automated patent classifiers are applied to a huge number of patent applications, which are then inspected by patent examiner to check the proof for the classification to make final classification decision. This is especially true for classification predictions that have low confidence by the classifiers. Due to this special requirement, high-performance patent classifiers that can explain their classification with extracted keywords, ready for quick inspection by the patent examiner, are strongly desirable.

Compared to other scientific and technological literature, patent texts have some unique characteristics. For example: the unregistered new technical terms frequently appear in the patent text, while the technical terms are a key component of patent documents, describing the most important knowledge in a specific domain. Moreover, the patent documents concentrate on particular fields, so some technical terms only appear in a specific domain but rarely in other fields. Besides, the technical terms also have the sparsity issue. Some terms appear less than five times in our training corpus. In addition, the format of patent description is more specific and rigorous than other scientific texts. The patent documents demand much more rigorous usage of language and text expression, due to certain criteria for patent applications imposed by patent offices. Meanwhile, it contains a wide range of detailed domain knowledge. With the development of new technologies, new terms constantly keep emerging from new areas. For example, more and more new terms, such as “deep learning”, “convolutional neural network”, and so on, have appeared with the rapid development of artificial intelligence techniques. 

Previous studies have applied various advanced data analysis methods to extract technological information from patent documents for different purposes. Gerken and Moehrle [1] constructed a similarity matrix between patent texts to determine novelty in patents. A large number of algorithms have been proposed to analyze patent trends and forecast technological developments in a particular domain [2,3,4,5]. Patent analysis techniques for determining patent quality for Research And Development (R&D) tasks [6,7] and technological road mapping [8] have also been developed. Meanwhile, keyword extraction algorithms have received a lot of attention as a quick way to acquire meaningful information from unstructured text, which can help to achieve more effective patent mining [3,9,10].

In keyword-based patent mining, almost all approaches are based on the assumption that keywords can represent the corresponding patent document well [3,11,12]. Thus, the quality of advanced patent analysis heavily relies on the quality of extracted keywords. However, there are few studies that evaluated the performance of keyword extraction methods [13]. Although keyword extraction techniques have made great progress in the past 10 years and many new algorithms have been proposed [14,15,16], the performance is still not satisfactory. For example, the best performances achieved on SemEval-2010 [17] and Hulth2003 [18] are only 27.2% and 38.7% in precision aspect. Traditional manual methods have high accuracy, but they are not efficient enough. On the other hand, computer-aided automatic keyword extraction methods are efficient, but not accurate enough. At the same time, existing automatic keyword extraction algorithms still suffer from some issues such as redundant expression, polysemy, synonyms thesaurus updating dynamically, and interdisciplinary content complexity. Besides, the evaluation strategies heavily rely on manually assigned keyword datasets [16,17,18].

Based on the usage of extracted keywords, keyword extraction algorithms can be roughly divided into two categories: one type are algorithms for extracting semantic keywords to summarize corresponding text [17,19] and the other type are for extracting discriminative keywords to classify texts into categories [16]. Both tasks require that the extracted keywords can represent the document well. Otherwise, the reliability and performance of subsequent analyses will be affected, which in turn makes it hard to draw reliable insights from analysis results. Considering these issues, this paper examines the effectiveness of deep learning-based keyword extraction methods and proposes a keyword extraction method based on the Skip-gram [20,21,22] model to effectively extract keywords from patent text for patent classification. Skip-gram is a deep learning algorithm which can effectively encode words into real-valued, dense, and low-dimensional vectors, representing the semantic and syntactic relation between words. 

The main contributions of this paper are:

(1) We propose a distributed representation based Patent Keyword Extraction Algorithm (PKEA), which could effectively extract keywords from patent text for patent classification.
(2) We develop a method to extract representative keywords from patents, which are then used as the features of the patent text for high performance classification by Support Vector Machine (SVM) classifiers.
(3) We design an evaluation method to measure the importance of each extracted keyword using information gain, which provides an indirect way to evaluate the effectiveness of extracting meaningful keywords when human-annotated keywords are not available.
(4) We compared our PKEA algorithm with Frequency, Term Frequency-Inverse Document Frequency (TF-IDF), TextRank and Rapid Automatic Keyword Extraction (RAKE). The PKEA outperforms other peer algorithms in terms of achieving higher patent classification accuracies and higher performance in terms of matching extracted keywords with human-annotated ones.

The overall structure of this paper is as follows. Section 2 describes related works on keyword extraction and keyword-based patent analysis. In Section 3, we firstly present the overall research framework and propose the criteria to evaluate the quality of extracted keywords. Then, we propose a novel keyword extraction algorithm for patent text. The detailed description of the experimental dataset and results are described in Section 4. Finally, we draw conclusions about our works in Section 5.

## 2. Related Work

### 2.1. Keyword Extraction Methods

Keyword extraction has been studied by many researchers, which is fundamental for text retrieval, text summarization, and many other text mining tasks. Generally, based on whether a labeled corpus is needed, automatic keyword extraction approaches can be broadly categorized as supervised and unsupervised methods. The advantages and drawbacks of different keyword extraction algorithms are summarized in Table 1.

In supervised keyword extraction approaches, the keyword extraction task is treated as a binary classification problem. A classifier determines whether each word or phrase in the document is a keyword. Many commonly used classification algorithms have been tried, such as decision trees [23], Naive Bayes classifiers [24], Support Vector Machines (SVM) [19], maximum entropy models [25], hidden Markov models [26], conditional random field models [14], and so on. Witten et al. [24] proposed a simple and efficient key phrase extraction algorithm (KEA) based on the Naive Bayes algorithm. Zhang et al. [14] modeled the keyword extraction task as the string labeling field which used features of documents more sufficiently and effectively based on conditional randomness. Their experimental results demonstrated that the conditional random field model improved the keyword extraction performance compared to other machine learning methods such as SVM, linear regression models, and so on. 

The drawback of supervised keyword extraction approaches is the need for a labeled corpus. The quality of the training corpus directly affects the performance of the model, thus affecting the results of keyword extraction. Moreover, since there are few labeled corpuses available, the training set often needs to be tagged by users themselves. Manual tagging of high-quality keywords from text leads to a great deal of difference in the experimental data, which is also expensive, time-consuming, and error prone. Therefore, how to get a high-quality training set is the bottleneck of these approaches.

Unsupervised keyword extraction methods include linguistic analysis, statistical methods, topic methods, and network graph based methods. These methods are used to extract keywords from an unlabeled corpus. Compared to supervised approaches, the major advantage of unsupervised methods is that there is no need of a manually labelled corpus. 

Term Frequency-Inverse Document Frequency (TF-IDF) has been widely used for keyword or key phrase extraction. Juanzi et al. [27] proposed a TF-IDF based approach to extract keywords in Chinese news documents. The experimental results showed that the TF-IDF significantly outperformed baseline methods on accuracy and significantly improved the efficiency of news event detection. Wartena et al. [31] presented three statistical methods to improve the performance of keyword extraction which were based on TF-IDF . The k-bisecting clustering algorithm shows the capability to extract strongly relevant keywords from Wikipedia articles [32].

Inspired by the extensive application and great success of the PageRank algorithm in the information retrieval field, Mihalcea and Tarau proposed a graph-based method TextRank [28] which is similar to the PageRank algorithm, whose edges in the network have weights and are assigned by the PageRank algorithm. Similar to the PageRank algorithm, TextRank determines the importance of a word through the importance of related words, uses the PageRank algorithm to iteratively calculate the importance of each word in the network and then sort it by the word’s PageRank value to select top ranked words as keywords. However, the TextRank keyword extraction algorithm requires many iterations to calculate the PageRank values for each word, typically between 20 and 30 iterations. Because of the high computational complexity, this algorithm is rarely used in large-scale text keyword extraction tasks. Wang et al. proposed [18] an improved TextRank based on pre-trained word embeddings to extract and generate keywords from scientific publications. They found that added word embedding vectors as external knowledge for the graph-based algorithm could improve performance when compared to the original algorithm. In addition, they also pointed out that training the word embeddings over a particular domain might further improve the performance. Rose et al. [30] proposed a Rapid Automatic Keyword Extraction (RAKE) algorithm to extract key phrases from individual documents. The RAKE algorithm has better performance on long key phrase extraction compared to TextRank.

### 2.2. Keyword-Based Patent Analysis

Keyword-based analysis has been applied to a wide range of patent mining tasks. There are a set of previous studies concerning how to extract meaningful keywords when a text-mining approach is applied to the patent analysis domain. Most of them applied the keyword extraction tools to solve a certain problem. For example, patent automatic classification, technology subject clustering, technology evolution analysis, future technological trends analysis, technology forecasting, strategy technology planning, infringement detection, novelty detection, technological road mapping, and competitor analysis.

Technology evolution analysis, technology theme generation, technology breakthrough innovation, and technology transformation are important contents of patent mining. Numerous studies can be fulfilled by applying keywords-based patent analysis approach for technology evolution analysis and future technological trends analysis. Kim, et al. [11] studied the patent clustering and visualization method based on keywords for prediction of emerging technologies; Joung et al. [3] proposed technical keyword-based analysis to monitor emerging technologies based on the TF-IDF; Yoon, et al. [33] drew the roadmap of mobile phone technology evolution based on word co-occurrences analysis and morphological analysis of the patent text keyword; Lee, et al. [34] found that new technological opportunities can be identified by building a patent keyword evolution map; Wu et al. [5] proposed a weighted Keyword-based Patent Network (WKPN) approach applying to green energy technology field to explore technological trends and evolution of biofuels.

Another application scenario of using keywords for patent analysis is technology subject clustering. To cluster technical topics, one of the commonly used approaches is based on keywords. The purpose of patent technology clustering is to discover distribution of technology themes. On the one hand, numerous studies have been proposed based on the keyword approach. Tseng et al. [35] studied the technology subject clustering in patent analysis and summarized the procedure of keyword selection: weight calculation, similarity calculation and clustering algorithm selection, multi-step clustering, clustering cluster labels, further grouping the clustering results. On the other hand, some researchers focus on the study of the relationship between technical topics. Kim et al. [11] analyzed the keywords representing emerging technology, based on the number of keyword clustering distribution and the patent application time. Wang et al. [36] used keywords to cluster technologic topics, treated the co-occurrence keywords between different clusters as technical transition words. Yoon et al. [12] identified the promising patent to predict the latest technology trends based on multi-dimensional scale analysis and outlier detection.

Meanwhile, a few studies concentrated on different keyword extraction methods [10], while others tried to identify the most appropriate section for keyword extraction. Xie et al. [13] selected a series of keywords in different sections from Automotive Software (ASW) related patents to identify the most appropriate section for patent identification. They found that the description is a rather noisy source of information for patent identification and the most effective strategy for identifying patents is using the title, abstract and claims section to extract keywords. Noh et al. [37] proposed guidelines for selecting and processing keyword sets. They considered different sections of the patent, number of the words’ appearances, the number of extracted keywords and the standardization method four keyword extraction factors, and also evaluated the keyword extraction performance based on clustering analysis and entropy values. They found that the most effective keyword extraction strategy for patent research is selecting 130 words from the abstract section based on a TF-IDF algorithm and Boolean expression.

## 3. Methods

### 3.1. Overall Research Framework and Proposed Algorithm

**Figure 1:**
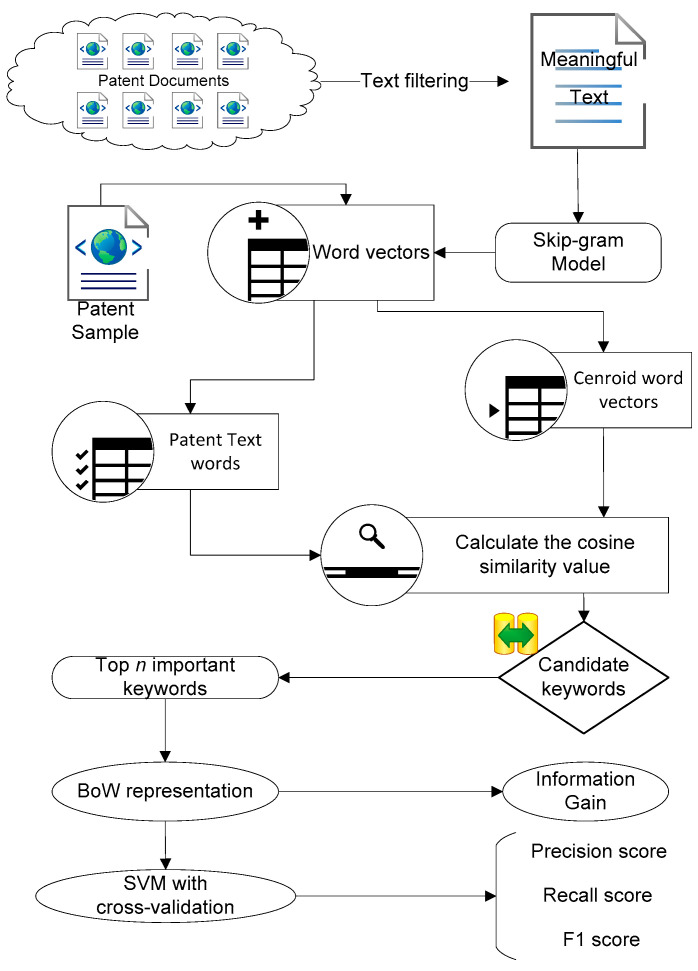
The overall process of keyword extraction and its evaluation measures.

Considering the preceding discussion and inspiration, we designed the research framework, which composes of patent keyword extraction algorithm and evaluation criteria. Figure 1 shows the overall process of keyword extraction and its evaluation criteria, and Algorithm 1 is the proposed patent keyword extraction algorithm based on distributed representation for patent classification (PKEA). 

In Figure 1, the Skip-gram model, *k*-means algorithm and cosine similarity are employed to build an effective keyword extraction algorithm. As we mentioned in Section 2.1, thoroughly evaluating the quality of extracted keywords is currently manual and intensive. To address this issue, we propose two evaluation criteria to automatically evaluate the quality of extracted keywords. 

In Algorithm 1, firstly, the Skip-gram model is applied to the training corpus to pre-train word embeddings. After the pre-training procedure, a *Word2VevTable* is obtained by applying the Skip-gram model that is detailed in Section 3.4. Secondly, for patents in each category, the corresponding words are converted to vectors, and then *k*-means algorithm (See Section 3.5) is applied to generate the current centroid vector *CentroidVector.* After obtaining the corresponding centroid vector, each candidate keywords list is converted to a vectors list. The similarity values between each candidate keyword to centroid vector is calculated by using cosine similarity function Cosine Similarity (See Section 3.6). Next, the top *n* keywords for each patent document are obtained by sorting the *KeywordsDict* by value. Finally, the keywords are extracted from patent texts by applying our PKEA.

**Algorithm 1** Patent Keyword Extraction Algorithm1: **Input:** Pre-defined category words list *WordsList*, Candidate keywords for each category list *CanWordsList*, Pre-trained word embedding *Word2VevTable*, Number of keywords *n*
2: **Output:** Ranked Keyword List *KeywordsList*
3: **for**
*i* = 0 **to** length(*WordsList*) **do**
4:   *WordVectorsList* = *Word2VevTable*[*WordsList*[*i*]]
5:   *CentroidVector* = *k*-means*(WordVectorsList*)
6:   **for**
*k* = 0 **to** length(*CanWordsList*[*i*]) **do**
7:    *CurrentWordsList = CanWordsList*[*i, k*]
8:    *CurrentWordVectorsList* = *Word2VevTable*[*CurrentWordsList*]
9:    *SimilarityValuesList* = CosineSimilarity(*CentroidVector*, *CurrentWordVectors*)
10:    *KeywordsDict* = Zip(*CurrentWordsList*, *SimilarityValuesList*)
11:    *RankedKeywordsDict* = SortByValue(*KeywordsDict*)
12:    *CurrentKeywordsList* = *RankedKeywordsDict*[*:n*]
13:    *Append*(*CurrentCategoryKeywordsList, CurrentKeywordsList*)
14:   **end for**
15:   *Append*(*KeywordsList*[*i*]*, CurrentCategoryKeywordsList*)
16: **end for**
17: *Output*(*KeywordsList*)

### 3.2. Information Gain Based Criterion

We propose two evaluation criteria to evaluate the performance of keyword extraction in each experimental case, both in macroscopic and microcosmic aspects. In order to evaluate the performances of various keyword extraction methods in the microcosmic aspect, we propose an evaluation method to measure the importance of each extracted keyword according to information gain theory. 

Documents from the same category have the same topic, thus they should have a similar set of keywords for discriminating between the other categories. To determine which keyword in a given set of documents is most useful for discriminating between the categories, we can calculate Information Gain (IG) to know how important an extracted keyword of the feature vector is. When it comes to IG, we have to talk about the concept of information theory and entropy. If an event $x_{i}$ occurred, then it contains the amount of information for:

(1) $$I(x_{i})=-\log_{2}p(x_{i})$$

If event $x_{i}$ does not occur, then the $I\left(x_{i}\right)$ indicates the uncertainty of the event. The essence of entropy is to measure the uncertainty of a system. The bigger the uncertainty is, the higher the entropy is. It is defined as the average amount of information for all events in a system, and can also be considered as the expectation of variable uncertainty. Given a system S is made up a series of variables $X=\left(x_{1},x_{2},x_{3},\ldots ,x_{n}\right)$, probabilities of occurrence are $p\left(x_{1}\right),p\left(x_{2}\right),p\left(x_{3}\right),\ldots ,p\left(x_{n}\right)$, then information entropy can be used to measure the amount of information in system S. The general formula of information entropy is:

(2) $$Entropy(S)=-\sum_{i=1}^{N}p(x_{i})\log p(x_{i})$$

The IG is a common approach for feature selection which reflects the gain of the whole system after added a new feature. 

(3) $$\begin{array}{l} Entropy(S|f_{new})\\ =-p(f_{new})\sum_{i=1}^{N}p(x_{i}|f_{new})\log p(x_{i}|f_{new})-p(\bar{f_{new}})\sum_{i=1}^{N}p(x_{i}|\bar{f_{new}})\log p(x_{i}|\bar{f_{new}}) \end{array}$$

Here the $p\left(f_{new}\right)\;$ denotes the probability of a new feature appearing in the samples, the $p\left(\bar{f_{new}}\right)\;$ means the probability of a new feature not appearing in the samples. The greater the IG is, the more important the new feature is. Then, we can judge the contribution of the feature to the classification system through the IG. The general formula for IG is as follows:

(4) $$Gain(f_{new},S)=Entropy(S)-Entropy(S|f_{new})$$

We can obtain the importance of each keyword by calculating $IG_{score}$. The higher total $IG_{score}$ is, the better performance of the keyword extraction algorithm is. For some texts in particular domains which do not exist human annotated keywords. In this case, the information gain in our paper can be used as an auxiliary evaluation measure to show that our algorithm can extract meaningful keywords. 

### 3.3. SVM Classification-Based Criterion

From the macro point of view, entire extracted keywords are treated as the features which represent the overall patent text. A SVM classifier with linear kernel is used to evaluate whether keywords could represent the overall patent text. The patent documents are represented by a set of keywords, so we can conduct a series of classification experiments using keywords as input features to classify each patent text into the corresponding category. To evaluate the result of each experiment, we use the most popular evaluation metrics, as follows. 

We can calculate $\;Precison_{score}$, $\;Recall_{score}$, and $F1_{score}$ for each prediction. The precision score is the number of correct predictions divided by the number of all returned predictions.

(5) $$Precison_{score}=\frac{correct\;predictions}{all\;predictions}$$

The recall score is the number of correct predictions divided by the number of all relevant patent documents.

(6) $$Recall_{score}=\frac{correct\;predictions}{all\;relevant\;patent\;documents}$$

(7) $$F1_{score}=2\times \frac{Precison_{score}\times Recall_{score}}{Precison_{score}+Recall_{score}}$$

The $\;Precison_{score}$, $\;Recall_{score}$, and $F1_{score}$ are denoted as Precision, Recall, and F1 respectively.

### 3.4. Skip-Gram Model for Patent Text Representation 

**Figure 2:**
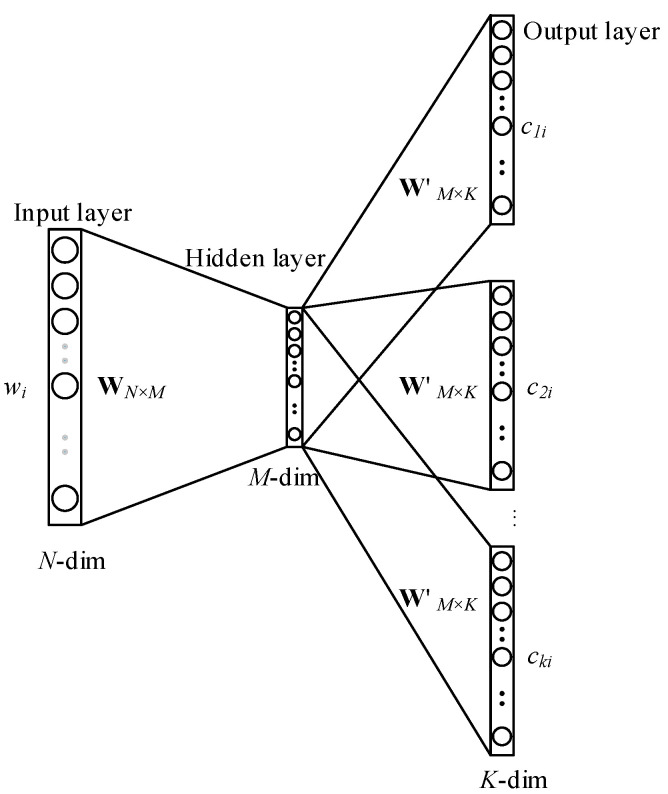
The architecture of Skip-gram model [20].

A word representation method deals with how to represent words by continuous vectors. There is a long history of representation of words as continuous vectors. Y. Bengio et al. [38] proposed a very popular model to estimate a neural network language model (NNLM), which consists of a feed-forward and back-propagation neural network. The former neural network includes a linear projection layer and a nonlinear hidden layer. The latter neural network is used to train a statistical language model that learns to map words into vector representations. In this paper, we employ the Skip-gram model proposed by Mikolov et al. [20,21] as our distributed word representation approach. This model is based on the distribution hypothesis that words in similar contexts have similar meanings. It has capability of learning high-quality word vectors from unstructured text data with billions of words, and with millions of words in the vocabulary. The most important thing is that after the training procedure each word gets the corresponding word vector which can be considered as the projection of the word in a syntactic and semantic space. Figure 2 shows the architecture of the Skip-gram model.

In this model, $w_{1},w_{2},w_{3},\ldots ,w_{n}$ are the training words, and $c_{1},c_{2},c_{3},\ldots ,c_{n}$ denote their context, which can be generated according to the center word $\;w_{i}$. k represents the number of context words. The word-context dependency relationship can be represented by a conditional probability  p. The goal of the Skip-gram model is to maximize the average log probability:

(8) $$\max(\frac{1}{n}\overset{n}{\underset{i=1}{\sum }}\underset{-k\leq j\leq k,j\neq 0}{\sum }\log(p(c_{i+j}|w_{i})))$$

A larger *k* result in a larger context and thus can lead to higher accuracy [39]. It also costs more time to train. When the probability p is put into the softmax function, we get:

(9) $$p\left(c|w;\theta \right)=\frac{e^{v_{c}\cdot v_{w}}}{\sum_{c^{\prime }\in C}e^{v_{c^{\prime }}\cdot v_{w}}}$$

where C is the vocabulary set of the context, W is the set of training words $w_{1},w_{2},w_{3},\ldots ,w_{n}$, D is the set of C and W. $v_{w}$ and $v_{c^{\prime }}$ are the “input” and “output” vectors represented of w. Put probability (9) into the objective function (8), we have:

(10) $$\max(\underset{\left(w,c\right)\in D}{\sum }\log\left(p\left(c|w\right)\right))=\max(\underset{\left(w,c\right)\in D}{\sum }(\log e^{v_{c}\cdot v_{w}}-\log\underset{c^{\prime }}{\sum }e^{v_{c^{\prime }}\cdot v_{w}}))$$

But calculating objective function (10) is non-trivial because of the computing cost since log(p( c|w;θ)) is proportional to W, which is often large. To address this issue, negative-sampling can be used to reduce the cost of computation. The main idea of negative-sampling is optimizing a different objective function. As mentioned earlier, D is the set of random (w,c) pairs that are all correct. Correspondingly, we can generate $D^{\prime }$ as the set of random (w,c) pairs that are all incorrect. Then the optimization objective function becomes:

(11) $$\max(\underset{\left(w,c\right)\in D}{\sum }\log\frac{1}{1+e^{-v_{c}\cdot v_{w}}}+\underset{\left(w,c\right)\in D^{\prime }}{\sum }\log\frac{1}{1+e^{v_{c}\cdot v_{w}}})$$

Let $\delta \left(x\right)=\;\frac{1}{1+e^{-x}}$ then the objective function (11) can be expressed as:

(12) $$\max(\underset{\left(w,c\right)\in D}{\sum }\log\delta (v_{c}\cdot v_{w})+\underset{\left(w,c\right)\in D^{\prime }}{\sum }\log\delta (-v_{c}\cdot v_{w}))$$

Compared to the objective (10), we can easily find that objective (12) will offset cumulative items. Thus the computational complexity will be significantly reduced. 

After the training process, the word embeddings are obtained, which encode the semantic and syntactic information in to real-valued, dense and low-dimensional vectors. In this paper, we set the hyper-parameters as follows: The minimum word count, window size and dimension of embedding vector in the PKEA algorithm is set to 3, 5 and 200 respectively. 

### 3.5. Using k-Means to Find Centroid Words

The *k*-means algorithm is a commonly used clustering algorithm. It is a kind of partitional clustering algorithm. The basic idea is to partition the given data into k clusters. Given a set of vectors $V=\;\left(v_{1},v_{2},v_{3},\ldots ,v_{m}\right)$, which belongs to k categories, the Euclidean distance between points p and q is defined as follow:

(13) $$d_{\;Euclidean}(p,q)=\sqrt{{\left(p_{1}-q_{1}\right)}^{2}+{\left(p_{2}-q_{2}\right)}^{2}+\ldots +{\left(p_{n}-q_{n}\right)}^{2}}=\sqrt{\sum_{i=1}^{n}{\left(p_{i}-q_{i}\right)}^{2}}$$

Choose k (random) data points (seeds) to be the initial centroids, cluster centers. The total distance will be:

(14) $$D_{whole}=\sum_{i=1}^{k}\sum_{v\in V}d_{{}_{\;Euclidean}}(v,s_{i})$$

Formally, the objective is to find:

(15) $$argmin\sum_{i=1}^{k}\sum_{v\in V}d_{\;Euclidean}(v,s_{i})$$

where $s_{i}$ is an initial centroid point. To minimize the objective, the *k*-means algorithm works as follows: Firstly, assign each data point to the closest centroid. Then, re-compute the centroids using the current cluster memberships. If a convergence criterion is not met, repeat the last two steps. This process continues until the centroids settle down and stop moving, after which the clustering is complete.

### 3.6. Finding Keywords by Calculating the Similarity

As mentioned above, we already have trained the word vector for each word and generated the centroid word for each patent category. For each $w_{ij}$ in document $d_{i}$, we calculate the cosine similarity between the word vectors $w_{ij}$ to the current centroid word vector $s_{i}$ as below:

(16) $$Sim(w_{ij},s_{i})=cos(w_{ij},s_{j})=\frac{w_{ij}^{T}\cdot s_{i}}{\left||w_{ij}||||s_{i}|\right|}$$

After calculating the cosine similarity, we get similarity value list for each document $d_{i}$. Then the similarity values are sorted from largest to smallest. Therefore, the extracted keywords for each document are the top n words which have largest similarity values with current centroid word. 

## 4. Comparison Experiments Results and Analysis

In this section, we describe details of the experimental dataset and presented the experimental results. The main goal is to validate whether our proposed algorithm could effectively extract keywords from patent text. In Section 4.1, we give a detailed description of our experiment dataset. In Section 4.2, we report the experimental results carried out by our PKEA and the baseline keyword extraction algorithms.

### 4.1. Test Datasets

In order to check the performance of PKEA, we used a benchmark dataset and a designed dataset.

**Autonomous cars patent corpus.** The experiment corpus in this paper is collected from Google Patent. We collect five distinct categories of patent documents which are related to autonomous cars. The corpus includes GPS systems, lidar systems, object recognition systems, radar systems and vehicle control systems, with 500 documents in each category. A patent document usually includes meta-data information and narrative text. The document number, issued date, patent type, classification information, inventors and applicant companies or individuals belong to meta-data information. The narrative text consists of abstract, claims, and description section.

More specifically: the title of a patent indicates the name of the patent; the abstract part gives a brief technical description of the innovation; the patent type explains patent’s type, and the classification part presents one or multiple class labels. The claim section’s main function is to protect the inventors’ right without any detailed technical information. The description section describes the process, the machine, manufacture, composition of matter, or improvement invented, a brief summary and the background of the invention, the detailed description, and a brief description of its application. The documents also contain meta-information on assignee, date of application, inventor, and so on. We do not collect any meta-data in our dataset since we focus on extract keyword from text.

Noh et al. [37] conducted a series of experiments to evaluate the representativeness of a keyword set from different sections of patents. They found the description section has the highest entropy value possibility due to its “noisy” words. After they compared with three keyword extraction strategies found that extracting keyword from abstract section lead to the best result. Therefore, in this study, we use the abstract section from patent as our experiment corpus.

**SemEval-2010** [17] **dataset**. The SemEval-2010 dataset is a benchmark dataset in key phrases extraction filed which consist of 144 training and 100 test papers belonging to four 1998 ACM classification: C2.4 (Distribution System), H3.3 (Information Search and Retrieval), I2.11 (Distributed Artificial Intelligence) and J4 (Social and Behavioral Sciences). Each article has two types of key phrases assigned by author and reader. Table 2 shows the distribution of the number and key phrases in training and test dataset. 

### 4.2. Comparison Results and Analysis

In keyword extraction tasks, a user is often required to manually evaluate the algorithms’ performance. Usually, many evaluation methods need a manually-assigned keywords dataset to calculate the Precision, Recall and F1. However, in this study, we proposed two evaluation measures to evaluate the performance of our proposed algorithm and the other baseline methods, which are independent of a manually assigned keywords dataset.

Firstly, we list the top 10 keywords in each patent category which are extracted by our proposed algorithm in Table 3.

As we mentioned in Section 3.1, the $IG_{score}$ can represent the contribution of a keyword adding to the system. To illustrate the process of calculating IG for each keyword, we give five patent document samples with four keywords in Table 4**.** There two categories patents: A and B, each category consists of several documents and each document contains some keywords. We use 1 to denote if a keyword appeared in a document and use 0 to denote that the keyword did not appear in the document.

As it can be observed from the Table 1 and Table 2 of 5 documents belong to category A and Table 3 of 5 documents belong to category B, thus the initial entropy is calculated as follow:

(17) $$\begin{array}{l} Entropy{\left(S\right)}_{init}=-\sum_{i=1}^{N}p(x_{i})\log p(x_{i})\\ =-\frac{2}{5}\log_{2}\frac{2}{5}-\frac{3}{5}\log_{2}\frac{3}{5}=0.97 \end{array}$$

Next, we can calculate the entropy $Entropy(S|f_{GPS})$ as the Formula (3).

(18) $$\begin{array}{l} Entropy(S|f_{GPS})\\ =-p(f_{GPS})\sum_{i=1}^{N}p(x_{i}|f_{GPS})\log p(x_{i}|f_{GPS})-p(\bar{f_{GPS}})\sum_{i=1}^{N}p(x_{i}|\bar{f_{GPS}})\log p(x_{i}|\bar{f_{GPS}})\\ =-\frac{2}{5}(-\frac{2}{2}\log_{2}\frac{2}{2}-\frac{0}{2}\log_{2}\frac{0}{2})-\frac{3}{5}(-\frac{1}{3}\log_{2}\frac{1}{3}-\frac{2}{3}\log_{2}\frac{2}{3})\\ =0.55 \end{array}$$

Therefore, the IG of keyword *GPS* equals to $Entropy{\left(S\right)}_{init}$ minus $Entropy(S|f_{GPS})$, so the IG can be obtained as follow. 

(19) $$\begin{array}{l} Gain(f_{GPS},S)=Entropy{\left(S\right)}_{init}-Entropy(S|f_{GPS})\\ =0.97-0.55\\ =0.42 \end{array}$$

**Figure 3:**
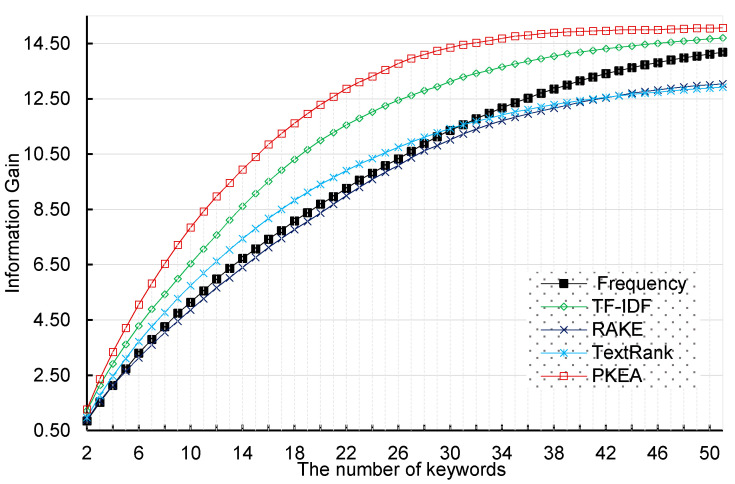
The sum $IG_{score}$ of entire keywords extracted by five algorithms.

For each extracted keyword, we can repeat the Formulas (5)–(7) to calculate IG for the classification system. In our experimental dataset, each category consists of 500 patent documents, hence our dataset has a discrete uniform distribution. We employed five keyword extraction algorithms to the corpus. Figure 3 illustrates the total $IG_{score}$ of entire keywords extracted by five algorithms.

For a fair comparison between five keyword extraction methods, we conduct each experiment ten times under the same conditions. We extract the same number of keywords for each patent in each experiment to calculate the $IG_{score}$ of entire keywords. From Figure 3, we can find that our PKEA obtained the highest $IG_{score}$ among all methods. This indicates that the PKEA can extract more representative words from the text than the others under the same conditions. Moreover, when we use PKEA to extract more than 35 keywords for a patent, the $IG_{score}$ barely increases. On the contrary, with the number of keywords increasing, the rest of approaches consistently improve $IG_{score}$. Furthermore, the TF-IDF-based and frequency-based approaches need to extract 50 keywords to reach the same score. It demonstrates that we can obtain a better result for a small number of keywords using our PKEA, thus it may benefit subsequent keyword related analysis tasks by reducing learning time and memory usage.

In addition, we also conducted a series of experiments using extracted keywords as the features to represent the corresponding patent. If the extracted keywords represent a patent’s overall text well, using keywords as input features for a classifier will lead to a high quality classification result. Based on this hypotheses, we designed a range of experiments using extracted keywords as input features to a classifier.

We chose Support Vector Machine (SVM) with a linear kernel as the classifier algorithm and used $\left\{K^{m}|m\in \left\{1,\ldots ,5\right\}\right\}$ to denote keywords set extracted from five algorithms. Then we randomly divided $K^{m}$ into 10 mutually exclusive equal sized subsets $\;\left\{S_{1},S_{2},S_{3},\ldots ,S_{10}\right\}$. In each experiment, we used $\left\{K^{m}-S_{i}\right\}$ as the training set, $S_{i}$ as the validation set and record the precision, recall and F1 score $\;p_{i}^{m},\;\;r_{i}^{m}$, and $\;f_{i}^{m}$, respectively.

**Figure 4:**
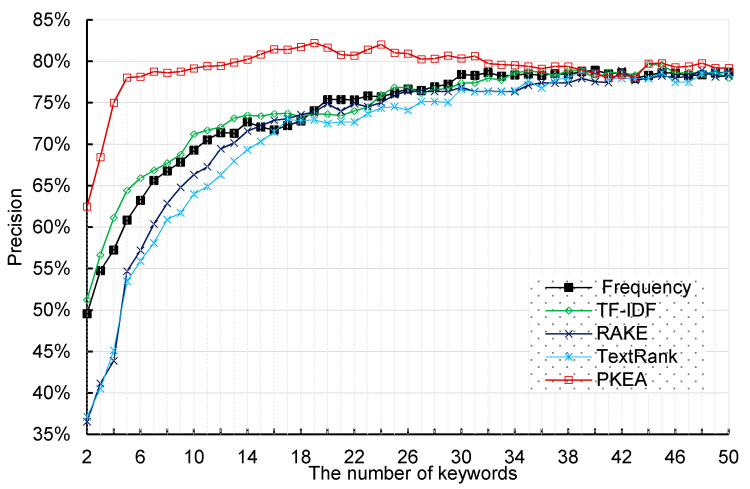
Precision scores obtained by Support Vector Machine (SVM) classifier using five keyword extraction algorithms.

In order to analyze the effect of different numbers of keywords on patent classification, different numbers of keywords ranging from 2 to 50 were extracted by 5 approaches. For each experiment, training and testing processes were repeated 10 times. Hence, we conducted 500 experiments which covered all configurations and the average precision scores under each situation are reported in Figure 4. Figure 4 shows average precision scores of the SVM classifier using a different number of keywords as input which are extracted by five kinds of keyword extraction algorithms. As can be observed in Figure 4, the highest precision score among all approaches is 81.61% which is obtained by our PKEA method. In addition, our PKEA achieved the highest precision scores under the circumstances of using the same number of keywords, except for using 50 keywords.

Moreover, when the input number of keywords is over 20, the PKEA method decreased precision score with increasing number of keywords. Meanwhile, as the keyword number grows, other approaches’ precision scores consistently improve. However, other approaches need to extract 50 keywords to reach approximately the same performance. This demonstrates that our PKEA method could use less keywords to represent the overall patent text. Therefore, we can infer that our PKEA has significantly improved the representativeness and quality of extracted keywords. 

**Figure 5:**
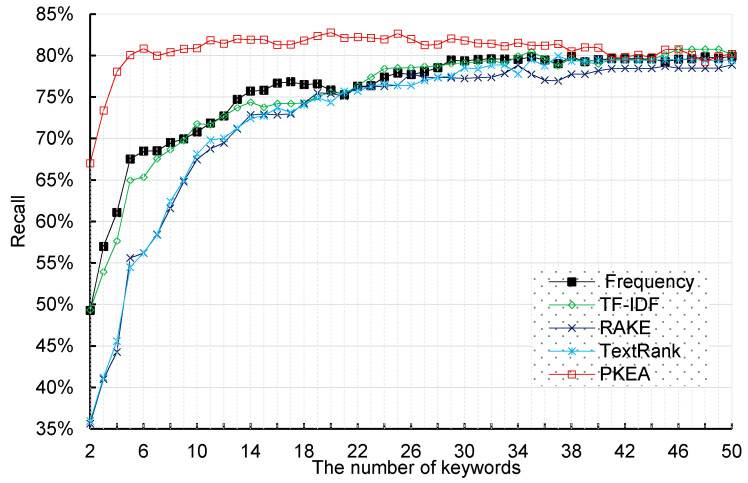
Recall scores obtained by SVM classifier using five keyword extraction algorithms.

Meanwhile, we also calculate the recall scores based on the classification results. Figure 5 shows the SVM classifier recall scores when using keywords extracted by these five keyword extraction algorithms as input features. The PKEA reaches 82.76% recall score when using 20 keywords. Compared to TF-IDF and frequency based approaches, our proposed method can use fewer keywords to better represent the corresponding patent text. Even only using 5 keywords, the Skip-gram-based algorithm achieves 81.05% recall score while the best performance achieve by other approaches is only 67.53%. This indicates that the PKEA has overwhelming advantage over other methods when extracting a small set of keywords. In other words, the PKEA has tremendously improved the quality of patent text keyword extraction in patent classification. 

**Figure 6:**
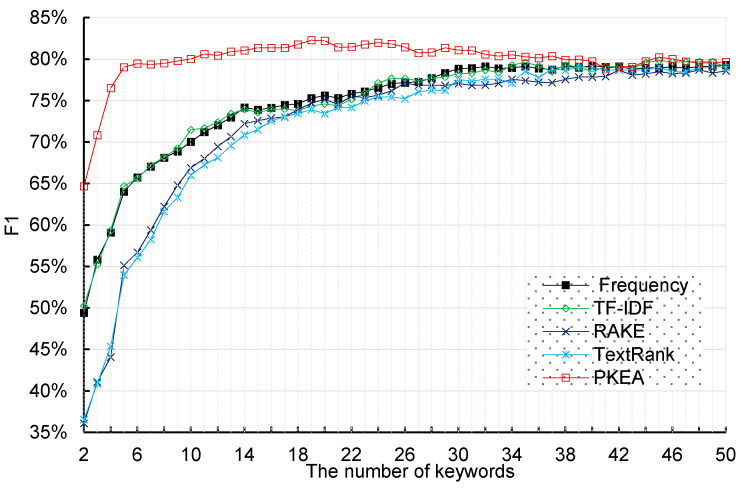
F1 scores obtained by SVM classifier using five keyword extraction algorithms.

Combining the precision and recall scores, we calculate the F1 scores for these five keyword extraction algorithms with different numbers of words to comprehensively understand the effects of word number on classification performance. As shown in Figure 6, we can find that the best classification performance 82.31% is achieved when the classifier uses 20 extracted keywords by our proposed method. While other algorithms only obtain 75.48%, 75.23%, and 73.42% respectively, under the same circumstance. The PKEA achieves the best performance in terms of compared metrics. The second highest performance is achieved by the TF-IDF algorithm. The worst results for the F1 metric is obtained by a RAKE-based approach. Besides, the figure about the number of keyword based comparisons clearly depict that the classification performance generally improves as the number of keywords increases. On the contrary, there is a subtle trend of decease for the number of keywords after value 20 for the PKEA approach. Nevertheless, F1 scores tend to converge as the total number of keywords increases form lower to higher number. This general trend can be explained as follows: it’s hard to represent the patent information when the number of keywords is too small, and thus classifiers’ performances are low. At this stage, increasing the number of keywords for a patent will bring great benefit to the classification performances. However, when the number of keywords reaches 40, the benefit of including more information is then balanced by the increased input dimension and computational complexity thus the classification performance tends to subtle decrease.

Furthermore, different approaches achieve the best performance under different conditions. For example, with 20 keywords, the PKEA method could achieve the best performance, while the TF-IDF-based method needs 35 keywords. To give a fair comparison across various algorithms, we choose the best performance achieved by different algorithms to represent their keyword extraction capabilities. Table 5 shows the best performance achieved by these five keyword extraction algorithms. Among all mean F1-scores presented in Table 5, the best performance of each algorithm are 81.99%, 78.72%, 79.72%, 78.51% and 79.05%, which are obtained by PKEA, Frequency, TFIDF, RAKE and TextRank respectively. Table 5 demonstrates that the mean F1-scores have many obvious differences between our PKEA and the other algorithms. Our PKEA improved the mean F1-scores compared to the other algorithms. 

In order to further evaluate whether our PKEA algorithm outperforms all the other explainable text classifiers in statistically significant way, we applied a paired-samples *T* test. We wanted to test if: $H_{0}:$ There is no significant difference between two sets of F1-scores. The results for 4 paired-samples *T* tests of F1-scores obtained by five keyword extraction methods are summarized in Table 6. On average, our PKEA has improved on the F1-scores of four baseline algorithms by 3%, 2.2%, 3.4% and 2.9% respectively. The standard deviations of F1-score differences are listed in the fourth column of Table 6. The 2-tailed *p*-values approach zero in each compared pair, which means that we should reject the $H_{0}$ since the *p* < 0.05 in each case. It can also be seen from Table 6, the *p* values indicate that the keyword extraction methods have significant effect on the F1-scores. 

Paper [17] provides SemEval-2010 dataset and evaluation methods. We apply our PKEA to the dataset and list comparisons of experimental results in Table 7. In the experiments on SemEval dataset, the F1-scores achieved by baseline approaches are around 10%, while our PKEA achieved decent performance, which outperformed three baseline methods for at least 2%, when we predict 5 key phrases for each article. However, the best performance on SemEval dataset is achieved by the Automatic Key Term Extraction from Scientific Articles (HUMB) algorithm, with the F1 scores of 19.8%, 26.0%, and 27.5%, which outperformed 19 participants. Our proposed method (PKEA) obtained decent but not the best performance, when compared with the best one, the HUMB algorithm. However, since the aim of our algorithm is to achieve high-performance patent classification with explainable keywords, it is satisfactory that our algorithm achieved comparably good performance in keyword extraction while achieving much better classification than these pure document-describing keyword extraction algorithms. In the SemEval task, algorithms are requested to extract key phrases from scientific articles with each key phrase containing 1 to 4 words and each category only consists of only around 40 articles in the training set. These factors led to the inferior performances of our PKEA compared to HUMB algorithm. Nevertheless, the experimental results on the SemEval dataset demonstrate that our PKEA can also extract meaningful key phrases from other types of texts other than patents. 

## 5. Conclusions

In this paper, we proposed an explainable high-performance keyword extraction algorithm PKEA, which exploits the capability of the Skip-gram model to capture the syntactic and semantics of words via its distributed representation. We evaluated our algorithm and other baseline algorithms over 2500 patent documents extracted from Google Patent. We compared PKEA with four of the most commonly used algorithms, including the simple term frequency and TF-IDF based baselines, the TextRank, and RAKE algorithms. 

We examined the effectiveness of these keyword extraction methods through two evaluation criteria tested over autonomous car related patents (GPS system, lidar system, object recognition system, radar system and vehicle control system) issued by the United States Patent and Trademark Office (USPTO). Firstly, we designed an evaluation method to measure the importance of each extracted keyword using information gain, which provides an indirect way to evaluate the effectiveness of extracting meaningful keywords when human-annotated keywords are not available. Secondly, a range of representative keywords have been extracted by five algorithms to validate which algorithm can achieve better performance. Then, the extracted representative keywords are used as the features of the patent text for high performance classification by SVM classifiers. Our results demonstrated that our PKEA algorithm is the most effective algorithm for extracting keywords from patent texts when extracting less than 20 words from the title and abstract section, as representative of the patent. Besides, the experimental results on the SemEval-2010 dataset al.so demonstrate that our PKEA has generalization capability to extract key phrases from the other types of texts. 

Several future studies are planned in our future works. One is to adding position features to train word embeddings. Moreover, generating key-phrases from patent texts is a crucial task to make our PKEA algorithm more useful in practical situations. We plan to design an improved PKEA algorithm which takes the position information of words into account and has the ability to generate key-phrases from the entire patent document.

## Figures and Tables

**Table 1:** Comparison of different keywords extraction approaches.

Categories	Methods	Advantages	Drawbacks	Application Scenarios
Supervised	Machine learning approaches (Decision Tree [23], Naïve Bayes [24], SVM [19], Maximum Entropy [25], HMM [26], CRF [14])	High readability, great flexibility to include a wide variety of arbitrary, non-independent features of the input. Can find several new terms which have not appeared in the training	Need labeled corpus	News, scientific articles, etc. Wildly applied.
Unsupervised	TF-IDF [27]	Without the need for a labeled corpus. Easy to implement, widely applied	Cannot extract semantically meaningful words. The keywords are not comprehensive. Not accurate enough.	News, scientific articles, etc. Wildly applied.
	TextRank [28]	Without the need for a labeled corpus. Has a strong ability to apply to other topic texts.	Ignored semantic relevance of keywords. The effect of low frequency keyword extraction is poor. High computational complexity.	Used in small-scale text keyword extraction task.
	LDA [29]	Without the need for a labeled corpus. Can obtain semantic keywords and solve the problem of polysemous. Easy to apply to various languages.	Prefer to extract general keywords which cannot represent the topic of corresponding text well.	Various languages.
	RAKE [30]	Without the need for a corpus. Very fast and the complexity is low. Easy to implement.	Cannot extract semantically meaningful words. Not accurate enough.	Extracting key-phrases from texts.
	PKEA (Our approach)	Can both extract semantic and discriminative keywords. Without the need for a corpus. Low computational complexity. High performance on extracting discriminative keywords. Easy to implement and apply to other type texts.	Need pre-defined category corpus.	Specially designed for extracting keyword from patent texts. Easy to extend to other scientific articles.

**Table 2:** Number of documents, author and reader-assigned key phrases in the training and test dataset.

Dataset	Number of Documents	Number of Categories	Number of Key Phrases		
			Author	Reader	Combined
Training	144	4	559	1824	2223
Test	100	4	387	1217	1482

**Table 3:** Top 10 keywords in each patent category extracted by the patent keyword extraction algorithm (PKEA).

Patent Categories	GPS System	Object Recognition	Vehicle Control System	Radar System	Lidar System
Keywords	GPS	Camera	Automobile	Radar	Lidar
	Satellite	Environment	Controller	Trajectory	Laser
	Altitude	Image	Communication	Operation	Detection
	Position	ORC	Assistance	Present-azimuth	Three-axis
	Synchronization	GUI	Speed	Prior-azimuth	Microwave
	Wavelength	Visibility	Guidance	Radiation	Receiver
	Telecommunication	Autonomous	Acceleration	Path	Luminescence
	Geo-mobile	Surrounding	Acquisition	Plurality	Reflection
	GPS-enabled	Video	Remote	Reference-location	Speedometer
	MS (communication device)	Multi-target	Roadway	Radar-sensor	Collision

**Table 4:** Keywords distribution in patent document.

Patent Documents	Keywords				Categories
	GPS	Image	Camera	Vehicle	
Patent 1	1	0	0	1	A
Patent 2	0	1	1	0	B
Patent 3	0	0	1	1	B
Patent 4	0	0	1	1	A
Patent 5	1	1	0	1	A

**Table 5:** Statistics of F1 achieved by fives algorithms.

Paired F1-Score Statistics					
		Mean	N	Std. Deviation	Std. Error Mean
Pair 1	PKEA	0.8199	10	0.03040	0.00961
	Frequency	0.7897	10	0.02141	0.00677
Pair 2	PKEA	0.8199	10	0.03040	0.00961
	TFIDF	0.7972	10	0.03500	0.01107
Pair 3	PKEA	0.8199	10	0.03040	0.00961
	RAKE	0.7851	10	0.03826	0.01210
Pair 4	PKEA	0.8199	10	0.03040	0.00961
	TextRank	0.7905	10	0.04965	0.01570

**Table 6:** Five algorithms paired F1 *T*-test results.

Paired F1-Score Test									
		Paired Differences					t	df	Sig. (2-Tailed)
		Mean	Std. Deviation	Std. Error Mean	95% Confidence Interval of the Difference				
					Lower	Upper			
Pair 1	PKEA-Frequency	0.03015	0.01052	0.00333	0.02263	0.03768	9.065	9	0.000
Pair 2	PKEA-TFIDF	0.02271	0.00878	0.00278	0.01642	0.02899	8.175	9	0.000
Pair 3	PKEA-RAKE	0.03482	0.01177	0.00372	0.02640	0.04325	9.354	9	0.000
Pair 4	PKEA-TextRank	0.02937	0.02150	0.00680	0.01399	0.04475	4.319	9	0.002

**Table 7:** Performance comparison between four algorithms on SemEval-2010 dataset.

Methods	Assigned by	Top 5 Candidates			Top 10 Candidates			Top 15 Candidates		
		Precision	Recall	F1	Precsion	Recall	F1	Precise	Recall	F1
TF × IDF [17]	R	17.8%	7.4%	10.4%	13.9%	11.5%	12.6%	11.6%	14.5%	12.9%
	C	22.0%	7.5%	11.2%	17.7%	12.1%	14.4%	14.9%	15.3%	15.1%
NB [17]	R	16.8%	7.0%	9.9%	13.3%	11.1%	12.1%	11.4%	14.2%	12.7%
	C	21.4%	7.3%	10.9%	17.3%	11.8%	14.0%	14.5%	14.9%	14.7%
ME [17]	R	16.8%	7.0%	9.9%	13.3%	11.1%	12.1%	11.4%	14.2%	12.7%
	C	21.4%	7.3%	10.9%	17.3%	11.8%	14.0%	14.5%	14.9%	14.7%
PKEA	R	20.0%	8.5%	11.9%	15.8%	12.7%	14.1%	13.2%	15.8%	14.4%
	C	24.6%	8.6%	12.8%	19.4%	12.9%	15.5%	16.1%	15.6%	15.9%
HUMB [17]	R	30.4%	12.6%	17.8%	24.8%	20.6%	22.5%	21.2%	26.4%	23.5%
	C	39.0%	13.3%	19.8%	32.0%	21.8%	26.0%	27.2%	27.8%	27.5%
